# Travelling Editorials

**Published:** 1843-06

**Authors:** 


### THE WESTERN JOURNAL.

Vol. VII.—No. VI.

LOUISVILLE, JUNE 1, 1843.

### TRAVELLING EDITORIALS.

We are frequently asked, whether the Western Journal is to be the vehicle of publication for the information we are collecting on the diseases of the West and South. To this we reply, that it is not; except when we can obtain manuscript communications, fitted by their authors for the press. Our design is to arrange and condense the whole into a separate work—a treatise—a book of home materials and home manufacture, intended for home consumption, and which, although it may be a homely production, will not be without its value to those for whom it is designed—the physicians of the country to which it relates, provided they communicate their observations freely, and have made them accurately.

*Congestive Fever.*

Since leaving the salubrious white sands of Pensacola Bay, whence we made our last communication, we have wandered among the tributaries of the Mobile—the great river of South Alabama, which is intersected with more navigable streams than any region we have visited. We have ascended the Alabama river four hundred miles, to its origin in the confluence of the Tallapoosa and Coosa; traversed those rapid and beautiful tributaries, and peeped into their cypress swamps and drowned lands; crossed the pine table lands which separate them from each other, and from the Tuscaloosa and Tombeckbee, with the broad and annually inundated alluvions of those navigable rivers; dipped into the eastern edge of the State of Mississippi; and snuffed up the exhalations of swamps, *sloughs, slashes,* hammocks, ponds, lagoons and lagging streams, in sufficient numbers to supply malaria for a continent, and raise a smile of complacent satisfaction on the lip of every advocate of the malarious origin of congestive fever; a disease which prevails, with some inequality, throughout all parts of the region in which we have spent the last month. In their history of the symptoms of this celebrated fever, the physicians, with whom we have conversed, have displayed a remarkable degree of coincidence; but their treatment, although in the main substantially the same, has been somewhat variant. One fact is unquestionable, that its mortality is chiefly referable to its not being treated at an *early* period. It is then, like epidemic cholera, a manageable disease. Time is its great ally, and delay renders it incurable. This fact is perfectly understood by the people, and still they too often postpone a resort to the profession; the reason of which is, perhaps, that, in its onset, it very generally assumes the mask of a harmless intermittent.

*Pneumonia.*

As the fever of last autumn subsided, a dangerous pneumonia arose, and has not yet entirely ceased. Its greatest prevalence was in the latter part of winter, and in early spring. On the whole, it has been as fatal as the fever which preceded it, and its treatment still more diversified and doubtful. In addition to the mortality it has already effected, it has laid the foundation of much *chronic* pulmonary disease, if we may judge from the fact that we have casually met with several patients, whose lungs were more or less hepatized. Pneumonia is a more constant winter disease in these latitudes, than is generally supposed; but it has not before been so frequent and fatal; which should no doubt be ascribed to the extraordinary coldness of the past winter and spring.

*Doctors, Calomel Doctors, and Botanical Doctors.*

These are the varieties into which the people of "these parts"

have divided, and the terms by which they designate, the men to whom they look for relief.

We are happy to observe that the first are far the most numerous; and although not to a great extent educated in our *young* Medical Institute, but in the schools of Charleston, Philadelphia, and Lexington, have many substantial claims to professional respectability; and would have still more, if they planted less cotton. We may, however, write with a partial pen, for their hospitality has every where been so great, that its acceptance has left us little time to look into their defects. Of calomel doctors we have seen but few, and those few have lost so much of the true physiognomy of the *caste*, as scarcely to be recognized. But our readers may ask, who, or what is meant in Alabama by a calomel doctor? We reply, he is, or rather *was*, a regularly educated physician, who assumed that calomel was the *only* remedy for malignant autumnal fever, and would certainly cure, if administered to the extent of a couple of ounces a day. Some of their patients occasionally had a pound *avoirdupois* in their stomachs at one time. But we do not propose to go into details, for the practice now sleeps with its victims; and many who once pursued it with unrelenting energy, at present unite with their brethren in its reprobation. A similar account may be given of drastic purging. A gentleman assured us that he had, under the direction of a physician, weighed out and administered to a fever patient, 1700 grains of calomel, and 2400 grains of aloes; and a physician informed us that he had given to a patient of the same class, 600 grains of a compound of equal parts of calomel, rheubarb and aloes, for six successive days. At present, as little calomel is prescribed in this country as in any part of the union, and perhaps less cathartic medicine. The ill success of the calomel and aloes doctors, brought into favor the botanical or steam doctors, whose methods were certainly preferable. Thus it is that eccentricity favors the growth of empiricism. Of the practice of these patentees, it is not our purpose to speak; but we cannot refrain from expressing regret, that any members of the medical profession should so far forget themselves as to hold consultation with men who are not educated to it, and who misrepresent and traduce it among the people, while they draw from its magazines all the means they employ, and use them in a perfectly arbitrary manner. Nor can we approve of the disuse of an old remedy by regular physicians, because it has been thus purloined and prostituted by reckless and unprincipled pretenders.

*Electro-Magnetism.*

In Tuscaloosa we were asked to look at a small electro-magnetic machine which an ingenious citizen of that place, Dr. Nelson Walkly, had constructed, and was applying to the cure of diseases. Dr. Walkly was an inquisitive and scientific mechanic, who turned his attention to medicine, which he studied for the purpose of making a systematic trial of electro-magnetism in the treatment of diseases. Within the last twelve or eighteen months, he has used it in chorea, epilepsy, neuralgia, palsy, chronic rheumatism, deafness, torpor of the liver with constipation, amenorrhœa, dysmenorrhœa, and several other complaints. Of his success and his failure, he gave us a detailed account, apparently with great candour; and we feel it a duty to say that he seems to have effected a cure, or afforded palliation, in several cases of those very intractable affections. It must certainly be admitted, that the profession has not yet made a full and fair trial of this agent, and we take great pleasure in commending Dr. Walkly's enterprise to the patronage of the physicians, and of the community in general, through this country.

*Dipping.*

The toilet-vocabulary of this country, has become enriched with the new and elegant word "dipping." A lady or a miss chews the end of a stick until she converts it into a kind of brush or fibrous mop, which she then proceeds to dip into snuff, with which she rubs her teeth and gums. At first she presses the powdered weed with a gentle hand, but becoming enamoured, at last touches so deeply as to consume a bottle of snuff in a week. Whole families and whole schools of girls are said, with a small number of cleanly exceptions, to be given to this method of titillating their nervous systems; and many, by the time they are full grown, have become so thoroughly impregnated with the powder, that their apparel might hang in a a hot room the whole summer, without being touched by the moths. We know of but two advantages from this habit. 1st. It may render them insensible to the breath of the other sex, who begin the use of tobacco with the study of grammar. 2d. It can be made a substitute for whiskey (now falling into discredit) by those who are in affliction. Thus we were told by a gentleman, that he lately saw a mother seated at the bed side of her expiring son, with an open dish of snuff on the table among his medicines, into which she plunged her "dipper" as often as she sighed; and when the tears of grief rolled down her cheeks, they mingled with streams of snuff-colored saliva from the corners of her mouth. It seems hard-hearted to condemn a custom fraught with such comforts, but we are compelled to say that it is not without many opposing effects. In our inquiries into the diseases of the sex in the south, we have already collected satisfactory evidence, that "dipping" is the cause of some and an aggravation of many more. We might refer to its effect on their breath, complexion, and cleanliness, but this we shall leave in the hands of the gentlemen who are immediately interested.

*Harmony in the Medical Profession.*

Our present pursuits afford us considerable insight into the state of the profession, and reveal, among other things, the social concord and discord of its members, in the same town or neighborhood. We are happy to say, that, with very few exceptions, we have found our brethren, in this quarter, rather more harmonious than we have ever seen them in any other part of the union. This does not arise from boyhood associations, in a common native land, for the communities of Mississippi and Alabama are really new; and their physicians are emigrants from several states, chiefly Georgia, the Carolinas, Virginia, Tennessee, and Kentucky. It therefore speaks well of them as men. Nor is it the fear of being challenged, keeping them in formal courtesy; for duelling is almost unknown and unthought of among them. On the contrary, we are disposed to ascribe much of it to an influence which equally condemns discord and duelling—that of christianity, under which we have found a great number of the most respectable and influential physicians. It is delightful to contrast this, with the infidelity, intemperance, and profanity, which prevailed thirty years ago. *Esto perpetua.*

*University of Alabama.—Geological Survey.—Medical Topography.*

No class of men have a deeper interest in Universities for classical and scientific elementary education, than physicians; for the profession can never attain its proper elevation among the callings of society, till those who study are well established in all preparatory branches of learning; which, at the present time, is far from being the case.

While in Tuscaloosa, we made a visit to the University, which is seated a mile in the country, and were introduced to its respectable Faculty. Professor Brumby, to whom is confided chemistry, mineralogy and geology, took us to his laboratory which is amply supplied with apparatus, and then to the library and cabinet. The former embraces three thousand four hundred and seventy-eight volumes, of which about one hundred and thirty relate to the natural sciences, while more than five hundred are arranged under the head of religion, although there is no professorship of theology in the University. It is a fair presumption that but few of these books are read by the students, and the proportion which they bear to the works on natural history, the natural sciences, anatomy, physiology, and medicine, is by far too great; especially when we consider that Professor Brumby, with a laudable zeal, attempts to teach more of these branches than are included in the title of his professorship, and is diligently engaged in adding to the cabinet, all sorts of mineralogical and zoological specimens. The Professor has also, with others, exerted himself on the Legislature to have a geological survey of the State undertaken, but as yet without success. Whenever it shall be accomplished, Alabama will be found second to no State in the Union, in the interest she will raise in the mind of the fossil zoologist, the botanist, the mineralogist, and the political economist. The southern half of the State is entirely tertiary, and abounds in the clays, sands, marls, and organic remains of that series of formations; then comes an extensive coal group, and after that, in the centre and more northern part, a tract of older rocks, highly metalliferous, where iron and gold—the most useful and the most precious of metals—have been already found in abundance. The flora of Alabama is equally rich and diversified with its geology, and comprehends, in great abundance, a variety of plants, useful in medicine and the arts, which might be made articles of exportation. Specimens of all its geological formations, with their organic remains and imbedded minerals; its plants and its animals, including those of its sea-shore; with analyses of its mineral waters, ought to be collected, and systematically arranged and preserved, in its State University. In the prosecution of this important enterprise, its medical topography would come to be understood, and means of improving the salubrity of unhealthy localities suggested. Were the University thus enriched, its ample endowment of $18,000 *per annum*, would enable the trustees to render it one of the most respectable institutions of learning and science in the Union; instead of dragging on with seventy-nine pupils, the present number. The sons of Alabama would then flock to it, and become enamoured with science, devoted to the study of their native land, and think of other and nobler things, in connexion with their native plains and hills and vallies, than mules and cotton.

*Hernando de Soto.—First Alabama Surgery.*

Travelling for the last month in the very region where De Soto, more than three hundred years ago, performed his wildest wanderings, we may be excused for referring to them. Entering the basin of Mobile river from the N. E., he struck the Tallapoosa, high up, and descended that river to the present Tallassee. Coming down upon the Alabama river, he fought the famous and ill-fated battle of Manvilla, about the junction of that river with the Tombeckbee, as some antiquaries conjecture, but obviously much higher up; whence he turned northwardly, and crossing the Tuscaloosa and Tombeckbee, proceeded towards the Mississippi river, in which he was finally buried. After the bloody battle of Manvilla, there were seventeen hundred wounds to be dressed. All the medicines and surgical *appareil* were burnt up; there was but one surgeon in the army, and he was inactive and inefficient. In this extremity, they made ointments of the fat of the dead Indians that lay around them; tore up their shirts for bandages; and enveloped the wounded limbs in their doublets; supporting the sick on broth made from the flesh of the slaughtered horses. Such was the first surgery on the banks of the beautiful Alabama, whose waters, after having long since washed away every vestige of Spaniard and Manvillian, continue to flow on, as pellucid as if they had never been dyed in blood.

*Greensboro', Ala.*, May 20, 1842. D.

